# How Owners of Epileptic Dogs Living in Italy Evaluate Their Quality of Life and That of Their Pet: A Survey Study

**DOI:** 10.3390/vetsci8080140

**Published:** 2021-07-23

**Authors:** Marisa Masucci, Valeria Di Stefano, Giulia Donato, Cyndi Mangano, Massimo De Majo

**Affiliations:** 1 Department of Veterinary Science, University of Messina, 98168 Messina, Italy; donatogiulia92@gmail.com (G.D.); mdemajo@unime.it (M.D.M.); 2 Clinica Veterinaria Clinivet, 98123 Messina, Italy; payval85@gmail.com; 3 Clinica Veterinaria Camagna, 89124 Reggio Calabria, Italy; cyndi_m@hotmail.it

**Keywords:** quality of life measuring, canine, seizure, antiepileptic treatment

## Abstract

Epilepsy is the most common chronic neurological disorder of dogs and requires a substantial commitment by the pet owner. The aim of this study was to evaluate how Italian owners of epileptic dogs receiving long-term treatment perceived their own quality of life (QoL) and that of their pet, using a list of key questions. A questionnaire was sent to owners of dogs affected by recurrent seizures and treated with antiepileptic drugs for at least three months. The questions included signalment, medical history and physical, social and psychological aspects associated with managing an epileptic dog. Eighty complete questionnaires were obtained. Most owners surveyed had a positive opinion on their dog’s QoL and they did not believe that commitment to managing their animals was a limitation of QoL. Dog QoL, seizure, frequency and severity were considered the most important factors in evaluating the efficacy of the antiepileptic treatment. The evaluation of the different aspects of QoL can help veterinary professionals understand the need for correct and exhaustive information provided to owners and the development of therapeutic plans and follow up, corresponding to the needs of dogs and owners.

## 1. Introduction

Epilepsy is one of the most common chronic neurological conditions in dogs. Its prevalence has been estimated to be 0.6–0.75% for idiopathic only or idiopathic and structural forms, respectively, in the general dog population [1,2].

Caring for an epileptic dog can be a source of stress and frustration. The administration of antiepileptic drugs (AED) requires perseverance and commitment, can be associated with side effects and requires regular veterinary checks [3,4]. Furthermore, treatment is unlikely to lead to complete remission of seizures and in the case of occurrence of status epilepticus the owner must administer additional emergency AED at home and/or urgently take the dog to the vet [3,4,5]. Therefore, managing an epileptic animal can be demanding in terms of responsibility and financial commitment and can have a strong emotional impact on owners, due to the paroxysmal manifestations of the disease and the recurrent and unpredictable nature of the seizures. Canine epilepsy can affect not only patients’ quality of life (QoL) but also owners’ QoL [6,7,8,9,10]. The QoL of both (dogs and owners) is often a key element in evaluating therapeutic success and deciding whether to continue treating the dog or to perform euthanasia [3,6,11].

Some human studies show that people living with an epileptic patient are at risk of developing post-traumatic stress disorder and having impaired QoL and psychological health [12,13,14]. Human QoL is defined by WHO as: “individuals’ perception of their position in life in the context of the culture and value systems where they live and in relation to their goals, expectations, standards and concerns” [15]. Therefore, it includes physical, psychological and social factors [15]. Many of these aspects are irrelevant and/or not evaluable for animals, so the assessment of QoL in these patients suffers from limitations and difficulties and cannot ignore the contribution provided by the owners. Although there is no consensus on its definition to date, studies have been carried out with the aim of selecting a list of key questions aimed at assessing the QoL for owners of dogs with idiopathic epilepsy [7,16].

Few studies have investigated the QoL in epileptic dogs and their carers, and all of them have been performed in the United Kingdom and the United States [3,6,9,17,18,19,20]. The aim of this study was to evaluate how Italian owners of epileptic dogs under long-term treatment perceived their own and their pet’s QoL through the use of a questionnaire.

## 2. Materials and Methods

Owners were selected from a population of clients of veterinary clinics in Sicily, Lazio and Emilia Romagna between October 2015 and April 2016. Inclusion criteria for dogs’ enrollment were: recurring epileptic seizures and antiepileptic treatment for at least three months. Considering that not all veterinarians involved were neurological specialists, it was not always possible to distinguish between idiopathic or structural epilepsy; therefore, etiology was not considered.

Dogs’ owners who met the inclusion criteria were invited by the veterinarian to fill in an anonymous questionnaire during the periodic check-ups or by telephone or by e-mail.

The questionnaire, formulated with partial modifications with respect to those proposed in the literature [3,6,7], consisted of 49 questions. The first nine concerned dogs’ signalment and medical history: breed, sex, age, weight, duration of the problem, age at time of the first seizure, type and dosage of AED, frequency of seizures. The next questions were divided into nine groups, concerning: pet–owner relationship (10–12), owner’s expectations on antiepileptic treatment (13–14), assessment of seizure control and emotional impact on the owner (15–19), AED side effects (20–30) [4,21,22,23], assessment of AED side effects and emotional impact on the owner (31–35), restrictions on the owner’s life (36–39), emotional impact of rectal diazepam use for emergency management of seizures at home (40), ideals in outcome assessment of seizures management (41–46), impact of caring for epileptic dog on the owner’s lifestyle (47–49) (Table 1).

Seizure frequency was scored between 1 and 8: more than one seizure per week (8), one seizure per week (7), three seizures per month (6), two seizures per month (5), one seizure per month (4), one seizure every 2 months (3), one seizure every 3–6 months (2), less than one seizure every 6 months (1). The answers to questions 10 to 49 were scored between 1 and 5: 1 for the answer “not at all”, 2 “a little”, 3 “enough”, 4 “a lot”, 5 “completely”.

### Statistical Analysis

Statistical analysis was performed by GraphPad Prism version 7.0 for Windows (GraphPad Software, San Diego, CA, USA). All data were subjected to the D’Agostino and Pearson normality test and, since almost all variables were not normally distributed, a non-parametric approach was used. Significance was set at *p* < 0.05. Total and median scores were calculated for each question (9 to 49).

To reduce the amount of data to be analyzed, composite scores were created by adding the score of conceptually similar questions within each group [24]. Cronbach’s alpha values were calculated to identify the degree of agreement between questions within a group. Cronbach’s alpha ≥ 0.7 was considered an acceptable reliability coefficient.

The Spearman rank correlation test was applied to evaluate correlations between questions. Correlations between the following questions were assessed, considering the critical values of the Spearman’s rank correlation coefficient (r) [25]:

- Age, duration of the problem, age at time of first seizure, number of AED used, dosage of AED (only for dogs treated with phenobarbital), and those concerning questions about: assessment of seizure control (composite score 15,17,19), emotional impact of seizures on the owner (composite score 16,18), AED side effects severity (composite score 20 to 30), acceptability of AED side effects (31), emotional impact of AED side effects on the owner (composite score 32 to 35), restrictions on the owner’s life (composite score 36 to 39), emotional impact of emergency management of seizures at home (40), ideals in outcome assessment of seizure management (composite score 41,42 and single score 43 to 46), impact of caring for the epileptic dog on the owner’s lifestyle (single scores 47 to 49);
- Frequency of seizures, relationship with the dog (10,11,12), expectations on treatment (13,14) and those of questions: assessment of seizure control (composite score 15,17,19), emotional impact of seizures on the owner (composite score 16,18), acceptability of AED side effects (31), emotional impact of AED side effects on the owner (composite score 32 to 35); restrictions on the owner’s life (composite score 36 to 39), emotional impact of emergency management of seizures at home (40), ideals in outcome assessment of seizure management (composite score 41,42 and single score 43 to 46), impact of caring for the epileptic dog on the owner’s lifestyle (single scores 47 to 49);
- AED side effects (composite score 20 to 30) and their emotional impact on the owner (31 and composite score 32 to 35).

## 3. Results

Eighty complete questionnaires were obtained from owners of as many epileptic dogs. Thirty-three (41%) were mixed-breed and 47 (59%) were purebred dogs (Table 2). Fifty-three (66%) dogs were males, and 27 (34%) were females. Age ranged from 18 to 180 months (median = 72) and weight from Kg 1.2 to 63 (median = 20). The duration of the problem ranged from 3 to 144 months (median = 24) and age of dogs at time of first seizure from 4 to 168 months (median = 30). Seizure frequency scores ranged between one and eight (median = 4) (Table 3). Most of the animals received phenobarbital (91%); moreover, 72.5% were treated with AED as mono-therapy and 27.5% received a multi-drug treatment (Table 3). Dosage of phenobarbital ranged from 1.7 to 20 mg/kg/day (median = 5.5).

Cronbach’s alpha values ≥ 0.7 were obtained for questions: 15,17,19 (alpha = 0.89), 16,18 (alpha = 0.81), 20–30 (alpha = 0.8), 32–35 (alpha = 0.85), 36–39 (alpha = 0.95) and 41,42 (alpha = 0.88). The scores of the questions from 10 to 49 are shown in supplementary file S1 and the significant correlations between the scores of the answers to the questionnaire are reported in Table 4, Table 5 and Table 6.

### 3.1. Pet––Owner Relationship

More than 70% of owners answered “enough”, “a lot” or “completely” to questions 10–12. They considered their dog like a child and able to understand their moods and problems and felt closer to it than to their friends or family.

### 3.2. Expectations of Antiepileptic Treatment

At the start of treatment, 77% of owners were moderately to fully optimistic about the possibility of improving their dog’s QoL (13) and 59% of them thought that therapy would require more effort (14). The scores of question 14 were positively and regularly correlated with the nuisance of AED administration (47) and positively and weakly correlated with the nuisance of veterinary checks (48) and seizure event (49) (Table 5).

### 3.3. Assessment of Seizure Control and Emotional Impact of Seizures on Owner

Eighty percent of owners believed that the seizures were completely controlled enough in the last three months, but almost all of them were generally concerned about their frequency and severity. The assessment of the success in seizure management (15,17,19) was weakly and positively correlated with the age at time of first seizure (6) and negatively and regularly correlated with the frequency of seizures (9) (Table 4 and Table 5).

### 3.4. AED Side Effects

AED side effects were, in decreasing order of severity: polyphagia, polyuria, polydipsia, somnolence, restlessness, weight gain, depression, ataxia, pruritus or erythema, diarrhea and vomiting. AED side effects’ severity (20–30) was weakly and negatively correlated with age (3) and regularly and negatively correlated with acceptability of AED side effects (31) (Table 4 and Table 6). On the other hand, there was a regular positive correlation between severity and emotional impact of AED side effects (32 to 35) (Table 6).

### 3.5. Assessment of AED Side Effects and Emotional Impact on the Owner

Ninety-one percent of owners considered the AED side effects in the last three months to be moderately to totally acceptable, about 30% considered them generally bothersome, 43% were concerned about physical effects and 31% about behavioral ones. There was a regular negative correlation between acceptability of the AED side effects (31) and their severity (20 to 30) (Table 6). Moreover, nuisance of and concern for AED side effects (32 to 35) were positively and weakly correlated with number of AED (7) and positively and regularly correlated with severity of AED side effects (20 to 30) (Table 4 and Table 6). Finally, there was a regular negative correlation between emotional impact of AED side effects (35 to 35) and dog’s age (3) and a weak negative correlation with age at time of first seizure (6) (Table 4).

### 3.6. Restrictions on the Owner’s Life

Dog epilepsy was a major problem in work, study or daily life for 42.5% of owners, and for an even smaller percentage, a limit on their social life (33.75%), free time (38.75%) and independence (36.25%). Limitations on the owner’s life (36 to 39) were regularly and negatively correlated with age of the animal (3) (Table 4). By contrast, there was a weak positive correlation between restriction on the owner’s life (36 to 39) and number of AED administered (7) (Table 4).

### 3.7. Emotional Impact of Rectal Diazepam Use for Emergency Management of Seizures at Home and Ideals in Outcome Assessment of Seizure Management

Administration of diazepam for emergency home seizures management caused varying degrees of concern (from “little” to “completely”) in 66.25% of owners. About 44% of them indicated that they were moderately to totally concerned about the endorectal administration of diazepam during the seizure.

### 3.8. Ideals in Outcome Assessment of Seizure Management

The most important elements in evaluating the success of antiepileptic therapy were, in decreasing order: the dog’s QoL, seizure frequency, seizure severity, AED side effects, influence on owner’s lifestyle and finally, cost of treatment. There was a weak negative correlation between the importance of seizure frequency and severity (41,42) and the seizure frequency (9).

### 3.9. Impact of Caring for the Epileptic Dog on the Owner’s Lifestyle

Only 20% of owners were moderately to totally bothered by drug administration and veterinary checks and 77.5% of them by the seizure event. There was a positive correlation between impact of caring for the epileptic dog on the owner’s life and perception of the need for greater commitment required by the treatment (14) (Table 5). The positive correlation was regular for nuisance of AED administration (47) and weak for nuisance of veterinary checks (48) and seizure event (49) (Table 5).

## 4. Discussion

This is the first study, conducted in Italy, which evaluated owners’ opinions about their own QoL and that of their dogs affected by epilepsy. Age and sex of the dogs evaluated as well as age of onset of seizure and duration of the problem are similar to those of previous studies performed in other countries [3,7,9,18,19].

The evaluation of the various aspects of the QoL is undoubtedly affected by the perceptions and motivations of the owners. Some factors were found to be able to influence them: expectations on treatment, age of the animal and age at time of the first seizure. Expectations on antiepileptic treatment influenced the impact of caring for the epileptic dog on the owner’s life. A previous study showed that regular contact with and easy access to the vet and thorough information regarding handling the animal during a seizure and therapeutic regime helped to reduce emotional stress associated with the dog’s epileptic condition [26]. As the dog’s age increased, the evaluation of AED side effects’ severity and their emotional impact on the owner tended to improve; moreover, the restrictions on the owner’s life were reduced. Furthermore, as age of seizure onset increased, assessment of the success in seizure management improved and emotional impact of the side effects of drugs tended to reduce, although weakly. In some human studies, the QoL improved with increasing age, age at seizure onset and duration of the problem [27,28,29], while in other cases the patient’s age and the duration of the problem [27,28] negatively correlated with the QoL. In both people and dogs, age is generally associated with an increased number of chronic health problems that contribute to worsening QoL; however, it also involves a decrease in expectations and a progressive adaptation to the compromised state of health [28,29]. In contrast to human studies, the duration of the problem did not affect any evaluation in our work. The dog was considered by the majority of owners as a child, family member or friend but the emotional bond with the animal did not affect any aspect of the QoL assessment.

In agreement with previous studies [9], the use of more AED tended to increase, although weakly, worry and annoyance and the limitations in the owner’s life. Drug resistance, which requires dose increases or combinations of AED, is frustrating and challenging to manage, both in terms of seizure control and increased side effects, and is an important cause of euthanasia in idiopathic canine epilepsy [6,7,9,30].

As already found in the literature [9,19], the importance attributed to frequency and severity of the seizures tended to reduce, although weakly, as the seizure frequency increased. Seizure frequency also affected the assessment of success in seizure management. According to a previous study, the frequency of seizures was not correlated with limitations in the owner’s life [6].

As already found by Lord and Podell [3], most owners found the AED side effects to be acceptable and not bothersome. Conversely, in the study of Chang and others and De Risio and others [6,18], the AED side effects were considered moderately to extremely bothersome by 58% of owners and were one reason for a decreased QoL. These differences could be, at least in part, due to the different treatments administered to the dogs in the different works: phenobarbital was the only AED administered in the study of Lord and Podell [3], while 61% of dogs in the study of De Risio and others [18] and 40% in that of Chang and others [6,18] received two or more AED. Most dogs in our study received only one AED (72.5%). The worsening of the side effects resulted in a reduction in their acceptability, increasing nuisance and concern. A correlation between side effects and poor QoL has been previously demonstrated by other authors [6,9,18,19].

A minority of owners reported significant limitations in their lives, according to Lord and Podell [3]. By contrast, in the study of De Risio and others [18], half of the owners felt that caring for epileptic dogs caused restrictions in their work, education, independence and social life. These differences could be due to the different characteristics of the groups under study, in terms of seizure frequency, form of epilepsy and number of AED administered. In the study of Lord and Podell [3], a low seizure frequency (≤1 per month) was found in the majority of dogs (63%) and De Risio and others [18] found a median frequency of five seizures per month [18]. In our study, the percentage of dogs with low seizure frequency was very similar (60%) to that of Lord and Podell and the median seizure frequency was lower (one seizure per month) than De Risio and others [3,18]. Furthermore, dogs with idiopathic and structural epilepsy were included in the study by Lord and Podell, while De Risio and others only considered subjects with idiopathic epilepsy [3,18]. In our study, no distinction was made between the two forms of epilepsy. Finally, the differences between the therapeutic regimes (mono- or poly-therapy) discussed above must be considered.

A minority of owners were moderately to completely concerned about the administration of diazepam for emergency home seizures management. We have not found the inverse association between QoL and administration of additional medications during the crisis that has been detected in previous studies [19].

According to the literature [6,19], the most important elements in evaluating the success of the therapy were: dog’s QoL, frequency and severity of the seizures and side effects of the treatment. By contrast, the impact on the owner’s lifestyle and the cost of seizure management were less important.

Failure to assess the type of epilepsy (structural or idiopathic) and seizures (single, cluster, status epilepticus; focal or generalized), elements that could have an influence on the QoL of the dogs and owners, constitutes a limitation of the present study. In fact, it has already been shown that dogs with cluster seizures have a worse QoL than those with single seizures [19] and that the risk of euthanasia increases in dogs with cluster seizures, status epilepticus and structural epilepsy [30,31].

## 5. Conclusions

Most owners had a positive opinion on their dog’s QoL and did not consider the commitment to manage the animal as a limitation of their own QoL. Moreover, dog’s QoL was considered the most important factor in evaluating the success of seizure management. The evaluation of the different aspects of the QoL is a fundamental element for the management of canine epilepsy by the veterinarian, who must provide correct and exhaustive information to the owner and develop treatment and monitoring plans that take into account the needs of the dog, considering at the same time the needs of the owner.

## Figures and Tables

**Table 1:** Questions 10 to 49 of the questionnaire.

Pet–Owner Relationship
10. Do you consider your dog like a child?
11. Do you think your dog understands your mood and your problems?
12. Do you feel closer to your dog than to your friends or family members?
Expectations on Antiepileptic Treatment
When you started treatment
13. Were you optimistic about the possibility of improving the dog’s QoL?
14. Did you think it would involve more effort?
Assessment of Seizures Control and Emotional Impact on the Owner
15. Has the frequency of seizures been acceptable in the last 3 months?
16. How worried are you about the frequency of seizures?
17. Has the severity of seizures been acceptable in the last 3 months?
18. How worried are you about the severity of seizures?
19. Overall, has the seizure management been successful in the last 3 months?
AED Side Effects
How severe have the following side effects been in the last 3 months?
20. Eating increase
21. Gaining weight
22. Drinking increase
23. Urinating increase
24. Sleeping increase
25. Depression
26. Restlessness
27. Lack of coordination when walking
28. Itchiness or skin rash
29. Vomiting
30. Diarrhea
Assessment of AED Side Effects and Emotional Impact on the Owner
31. Overall, have the side effects of the therapy been acceptable in the last 3 months?
32. How bothersome are the physical effects of the therapy (eating increase, gaining weight, drinking increase, urinating increase, lack of coordination when walking, itchiness, skin rash, vomiting, diarrhea)?
33. How bothersome are the mental effects of the therapy (sleeping increase, depression, restlessness)?
34. How worrying are the physical effects of therapy?
35. How worrying are the mental effects of therapy?
Restrictions on the Owner’s Life
36. In the past 3 months has your dog’s epilepsy caused conflict with your work, education or day-to-day activities?
37. In the past 3 months has your dog’s epilepsy limited your social life?
38. In the past 3 months has your dog’s epilepsy limited your free time?
39. In the past 3 months has your dog’s epilepsy limited your independence?
Emotional Impact of Rectal Diazepam Use for Emergency Management of Seizures at Home
40. Are you worried when you need to give rectal diazepam?
Ideals in Outcome Assessment of Seizure Management
How important are the following factors to you when assessing the outcome of seizure management?
41. Seizure frequency
42. Seizure severity
43. Side effects of AED
44. Dog’s QoL
45. Influence on your lifestyle
46. Cost of the seizure management (drugs, diagnostic procedures, monitoring)
**Impact of Caring for the Epileptic Dog on the Owner’s Lifestyle**
47. The administration of the medication is a nuisance?
48. Veterinary checks are a nuisance?
49. Seizure event is a nuisance?

**Table 2:** Dog breeds under study.

Breed	Number of Dogs
Akita Inu	1
Toy Poodle	2
Beagle	6
Border Collie	3
French Bulldog	1
English Bulldog	1
Pug	1
Cavalier King Charles Spaniel	1
Chihuahua	1
Cocker Spaniel	2
Corso	1
Dogo Argentino	1
Dogue de Bordeaux	1
Golden Retriever	2
Jack Russell	2
Labrador Retriever	4
Maltese	1
Mixed-Breed	33
Australian Shepherd	7
Caucasian Shepherd	1
Pekingese	1
Pinscher	1
Pit Bull	1
Rottweiler	1
Shar Pei	1
Volpino Italiano	1
Yorkshire Terrier	2

**Table 3:** Seizure frequency and treatments of the dogs under study. CLZ: clonazepam; GBP: gabapentine; IMP: imepitoin; KBr: potassium bromide; LEV: levetiracetam; PB: phenobarbital; PRM: primidone; AED: antiepileptic drugs.

	Number of Dogs
Seizure Frequency	
Less Than One Every 6 Months	10
One Every 3–6 Months	14
One Every 2 Months	7
One per Month	17
Two per Month	18
Three per Month	5
One per Week	1
More Than One per Week	8
AED	
PB	53
PB + KBr	11
IMP	4
PB + LEV	2
PB + KBr + LEV	2
KBr	1
IMP + LEV	1
PB + PRM	1
PB + KBr + IMP	1
PB + KBr + GBP	1
PB + KBr + CLZ	1
KBr + LEV + PRM	1
PB + KBr + LEV + GBP	1

**Table 4:** Correlations between the values of age (months), age at time of first seizure (months), number of AED (antiepileptic drugs) and the composite scores attributed to the answers of the questionnaire concerning: assessment of seizure control, severity of AED side effects, emotional impact of AED side effects, and restriction on the owner’s lifestyle. The values of r (Spearman rank correlation coefficient) and *p* (probability value) are reported, indicating positive correlations in green and negative correlations in yellow. NS: not significant.

	3. Age	6. Age at Time of First Seizure	7. Number of AED
15,17,19. Assessment of the Success in Seizure Management	NS	r = 0.3151 *p* = 0.0047	NS
20–30. Severity of AED Side Effects	r = −0.2899 *p* = 0.0091	NS	NS
32–35. Nuisance and Concern for AED Physical and Mental Side Effects	r = −0.4487 *p* < 0.0001	r = −0.3847 *p* = 0.0005	r = 0.3473 *p* = 0.0016
36–39. Restrictions on the Owner’s Life	r = −0.4319 *p* < 0.0001	NS	r = 0.3729 *p* = 0.0007

**Table 5:** Correlations between the scores attributed to seizure frequency and expectations on antiepileptic treatment and those attributed to answers of the questionnaire concerning: assessment of seizure control (composite score), importance of seizure frequency and severity (composite score), impact of caring for the epileptic dog on the owner’s lifestyle. The values of r (Spearman rank correlation coefficient) and *p* (probability value) are reported, indicating positive correlations in green and negative correlations in yellow. AED: antiepileptic drugs; NS: not significant.

	9. Seizure Frequency	14. Perception of The Need for Greater Commitment Required by the Treatment
15,17,19. Assessment of the Success in Seizure Management	r = −0.4644 *p* > 0.0001	NS
41,42. Importance of Seizure Frequency and Severity in Outcome Assessment of Seizure Management	r = −0.2633 *p* = 0.0183	NS
47. Nuisance of AED Administration	NS	r = 0.42 *p* = 0.0001
48. Nuisance of Veterinary Checks	NS	r = 0.3125 *p* = 0.0048
49. Nuisance of Seizure Event	NS	r = 0.2678 *p* = 0.0163

**Table 6:** Correlations between the scores attributed to the answers concerning AED (antiepileptic drugs) side effects severity (composite score) and those attributed to AED side effects assessment and emotional impact (composite score). The values of r (Spearman rank correlation coefficient) and *p* (probability value) are reported, indicating positive correlations in green and negative correlations in yellow.

	31. Acceptability of AED Side Effects	32 to 35. Nuisance and Concern for AED Physical and Mental Side Effects
20 to 30. Severity of AED Side Effects	r = −0.4096 *p* = 0.0002	r = 0.5901 *p* < 0.0001

## Data Availability

The data presented in this study are not publicly available due to privacy restrictions.

## Supplementary Materials

The following are available online at https://www.mdpi.com/article/10.3390/vetsci8080140/s1, Table S1: Number and percentage of answers obtained for questionnaire questions 10–49, total and median score values for each question.
