# Supplementary material for: How Owners of Epileptic Dogs Living in Italy Evaluate Their Quality of Life and That of Their Pet: A Survey Study
Source: Vet Sci. 2021 Jul 23;8(8):140. doi: 10.3390/vetsci8080140 (PMC8402623; doi:10.3390/vetsci8080140)
Supplement: Supplementary file 1 [file vetsci-08-00140-s001.zip › vetsci-1227939-supplementary.pdf]

**Supplementary Table S1**. Number and percentage of answers obtained for questionnaire questions 10-49, total and median score values for each question.

| | **Not at all (score: 1)** | **A little (score: 2)** | **Enough (score: 3)** | **A lot (score: 4)** | **Completely (score: 5)** | **Total score** | **Median score** |
|---|---|---|---|---|---|---|---|
| Pet-owner relationship | | | | | | | |
| 10. Do you consider your dog like a child? | 6 (7.5%) | 12 (15%) | 32 (40%) | 21 (26.25%) | 9 (11.25%) | 255 | 3 |
| 11. Do you think your dog understands your mood and your problems? | 5 (6.25%) | 12 (15%) | 24 (30%) | 23 (28.75%) | 16 (20%) | 273 | 3 |
| 12. Do you feel closer to your dog than to your friends or family members? | 6 (7.5%) | 16 (20%) | 30 (37.5%) | 22 (27.5%) | 6 (7.5%) | 246 | 3 |
| Expectations on antiepileptic treatment | | | | | | | |
| When you started treatment | | | | | | | |
| 13. Were you optimist about the possibility of improving the dog's QoL? | 4 (5%) | 14 (17.5%) | 28 (35%) | 26 (32.5%) | 8 (10%) | 260 | 3 |
| 14. Did you think it would involve more effort? | 17 (21.25%) | 16 (20%) | 19 (23.75%) | 20 (25%) | 8 (10%) | 226 | 3 |
| Assessment of seizures control and emotional impact on the owner | | | | | | | |
| 15. Has the frequency of seizures been acceptable in the last 3 months? | 10 (12.5%) | 11 (13.75%) | 28 (35%) | 13 (16.25%) | 18 (22.5%) | 210 | 3 |
| 16. How worried are you about the frequency of seizures? | 0 (0%) | 2 (2.5%) | 11 (13.75%) | 44 (55%) | 23 (28.75%) | 328 | 4 |
| 17. Has the severity of seizures been acceptable in the last 3 months? | 6 (7.5%) | 14 (17.5%) | 26 (32.5%) | 17 (21.25%) | 17 (21.25%) | 265 | 3 |
| 18. How worried are you about the severity of seizures? | 1 (1.25%) | 4 (5%) | 13 (16.25%) | 39 (48.75%) | 23 (28.75%) | 319 | 4 |
| 19. Overall, has the seizure management been successful in last three months? | 4 (5%) | 12 (15%) | 27 (33.75%) | 13 (16.25%) | 24 (30%) | 281 | 3 |
| AED side effects | | | | | | | |
| How severe have the following side effects been in the last 3 months? | | | | | | | |
| 20. Eating increase | 17 (21.25%) | 20 (25%) | 22 (27.5%) | 18 (22.5%) | 3 (3.75%) | 210 | 3 |
| 21. Gaining weight | 26 (32.5%) | 34 (42.5%) | 13 (16.25%) | 5 (6.25%) | 2 (2.5%) | 163 | 2 |
| 22. Drinking increase | 17 (21.25%) | 24 (30%) | 25 (31.25%) | 10 (12.5%) | 4 (5%) | 200 | 2 |
| 23. Urinating increase | 14 (17.5%) | 27 (33.75%) | 18 (22.5%) | 17 (21.25%) | 4 (5%) | 210 | 2 |
| 24. Sleeping increase | 17 (21.25%) | 35 (43.75%) | 19 (23.75%) | 8 (10%) | 1 (1.25%) | 181 | 2 |
| 25. Depression | 31 (38.75%) | 32 (40%) | 14 (17.5%) | 3 (3.75%) | 0 (0%) | 149 | 2 |
| 26. Restlessness | 23 (28.75%) | 33 (41.25%) | 14 (17.5%) | 6 (7.5%) | 4 (5%) | 175 | 2 |
| 27. Not coordination when walking | 36 (45%) | 29 (36.25%) | 9 (11.25%) | 6 (7.5%) | 0 (0%) | 145 | 2 |
| 28. Itchiness or skin rash | 49 (61.25%) | 21 (26.25%) | 5 (6.25%) | 5 (6.25%) | 0 (0%) | 126 | 1 |
| 29. Vomiting | 68 (85%) | 10 (12.5%) | 2 (2.5%) | 0 (0%) | 0 (0%) | 94 | 1 |
| 30. Diarrhea | 61 (76.25%) | 17 (21.25%) | 2 (2.5%) | 0 (0%) | 0 (0%) | 101 | 1 |

| | | | | | | | |
|---|---|---|---|---|---|---|---|
| Assessment of AED side effects and emotional impact on the owner | | | | | | | |
| 31. Overall, have the side effects of the therapy been acceptable in the last 3 months? | 0 (0%) | 7 (8.75%) | 32 (40%) | 22 (27.5%) | 19 (23.75%) | 293 | 4 |
| 32. How bothersome are the physical effects of the therapy (eating increase, gaining weight, drinking increase, urinating increase, not coordination when walking, itchiness, skin rash, vomiting, diarrhea)? | 24 (30%) | 29 (36.25%) | 17 (21.25%) | 8 (10%) | 2 (2.5%) | 175 | 2 |
| 33. How bothersome was the mental effects of the therapy (sleeping increase, depression, restlessness)? | 27 (33.75%) | 28 (35%) | 17 (21.25%) | 5 (6.25%) | 3 (3.75%) | 169 | 2 |
| 34. How worrying are the physical effects of therapy? | 18 (22.5%) | 27 (33.75%) | 21 (26.25%) | 12 (15%) | 2 (2.5%) | 193 | 2 |
| 35. How worrying are the mental effects of therapy? | 26 (32.5%) | 29 (36.25%) | 14 (17.5%) | 9 (11.25%) | 2 (2.5%) | 172 | 2 |
| Restrictions on the owner's life | | | | | | | |
| 36. In the past 3 months has your dog's epilepsy caused conflict with your work, education or day-to-day activities? | 24 (30%) | 22 (27.5%) | 18 (22.5%) | 10 (12.5%) | 6 (7.5%) | 192 | 2 |
| 37. In the past 3 months has your dog's epilepsy limited your social life? | 32 (40%) | 21 (26.25%) | 8 (10%) | 14 (17.5%) | 5 (6.25%) | 179 | 2 |
| 38. In the past 3 months has your dog's epilepsy limited your free time? | 32 (40%) | 17 (21.25%) | 9 (11.25%) | 15 (18.75%) | 7 (8.75%) | 188 | 2 |
| 39. In the past 3 months has your dog's epilepsy limited your independence? | 26 (32.5%) | 25 (31.25%) | 6 (7.5%) | 17 (21.25%) | 6 (7.5%) | 192 | 2 |
| Emotional impact of rectal diazepam use for emergency management of seizures at home | | | | | | | |
| 40. Are you worried when you need to give rectal dizepam? | 27 (33.75%) | 18 (22.5%) | 19 (23.75%) | 9 (11.25%) | 7 (8.75%) | 191 | 2 |
| **Ideals in outcome assessment of seizure** management | | | | | | | |
| How important are the following factors to you when assessing the outcome of seizure management? | | | | | | | |
| 41. Seizure frequency | 2 (2.5%) | 3 (3.75%) | 11 (13.75%) | 39 (48.75%) | 25 (31.25%) | 322 | 4 |
| 42. Seizure severity | 2 (2.5%) | 3 (3.75%) | 14 (17.5%) | 35 (43.75%) | 26 (32.5%) | 320 | 4 |
| 43. AED side effects | 6 (7.5%) | 13 (16.25%) | 24 (30%) | 32 (40%) | 5 (6.25%) | 257 | 3 |
| 44. Dog's QoL | 1 (1.25%) | 3 (3.75%) | 7 (8.75%) | 32 (40%) | 37 (46.25%) | 341 | 4 |
| 45. Influence on your lifestyle | 7 (8.75%) | 21 (26.25%) | 23 (28.75%) | 23 (28.75%) | 6 (7.5%) | 240 | 3 |
| 46. Cost of the seizure management (drugs, diagnostic procedures, monitoring) | 11 (13.75%) | 25 (31.25%) | 20 (25%) | 18 (22.5%) | 6 (7.5%) | 223 | 3 |
| Impact of caring for the epileptic dog on the owner's lifestyle | | | | | | | |
| 47. Is the administration of the medication a nuisance? | 40 (50%) | 24 (30%) | 12 (15%) | 3 (3.75%) | 1 (1.25%) | 141 | 1,5 |
| 48. Are veterinary checks a nuisance? | 33 (41.25%) | 31 (38.75%) | 13 (16.25%) | 2 (2.5%) | 1 (1.25%) | 147 | 2 |

| | | | | | | | |
|---|---|---|---|---|---|---|---|
| 49. Is seizure event is a nuisance? | 9 (11.25%) | 9 (11.25%) | 10 (12.5%) | 27 (33.75%) | 25 (31.25%) | 290 | 4 |
